# FINANCIAL FORTITUDE: IMPACT OF CAREGIVING ACROSS GENERATIONS

**DOI:** 10.1093/geroni/igae098.3844

**Published:** 2024-12-31

**Authors:** Quynh Vo, Shikha Bhurtel, Janice Bell, Pauline DeLange Martinez, Robin Whitney, Heather Young

**Affiliations:** UC Davis, Elk Grove, California, United States; UC Davis, Davis, California, United States; UC Davis, Davis, California, United States; UC Davis, Sacramento, California, United States; San Jose State, San Jose, California, United States; UC Davis, Ashland, Oregon, United States

## Abstract

There is growing evidence of the economic impact of unpaid family caregiving on physical, mental, social and financial wellbeing. The financial burden of caregiving has not however been fully examined nationally by generation. To address this gap, we examined relationships between generation and selected measures of financial fortitude among caregivers. This cross-sectional study used the 2020 Caregiving in the U.S. Survey to examine any reported impact of caregiving on savings, debt, and bills among adult caregivers who cared for an older adult. The primary independent variable was generation categorized as millennial, Gen X, and Baby Boomer. Survey-weighted logistic regression was used to model outcomes as functions of generation, controlling for socio-demographic and caregiving variables. Compared to Baby Boomers, millennials had higher odds of reporting impact on savings (OR=1.8; p=.006; 95%CI [1.17, 2.70]), debt (OR=2.2; p< 0.01; 95%CI [1.45, 3.40]), and bills (OR=2.6; < 0.01; 95%CI [1.69, 4.24]). Generation X also had higher odds of reporting impact on savings (OR=1.6; p=.006; 95%CI [1.15, 2.3]) and debt (OR=1.4; p=.03; 95%CI [1.02, 2.17]). Caregivers with higher incomes reported lower odds of impacts on all outcomes while those supporting more activities of daily living and caregiving for longer hours had higher odds. Additionally, caregivers with college degrees reported lower odds of impacts on bills. These findings highlight the role of generational status, socio-demographic and caregiving factors on financial outcomes, emphasizing the need for targeted interventions and policies that provide financial support to younger caregivers.

